# Operational risk management during disasters: A case of South African tourism small businesses

**DOI:** 10.4102/jamba.v17i1.1761

**Published:** 2025-04-30

**Authors:** Wonder Mahembe, Ashley T. Mutezo

**Affiliations:** 1 Department of Finance, Risk Management and Banking, College of Economic and Management Sciences, University of South Africa, Pretoria, South Africa

**Keywords:** operational risk management, small businesses, tourism, COVID-19, disaster risks, disaster preparedness, disaster learning, enterprise resilience

## Abstract

Small businesses, which were disproportionately affected by the COVID-19 pandemic, often lack the resources for effective operational risk management (ORM), with existing frameworks like International Standardization Organization (ISO) 31000 proving too complex and resource-intensive. There is, however, still minimal research into ORM frameworks tailored to the needs of Small, Micro, and Medium Enterprises (SMMEs) facing disaster risks. This study developed and evaluated a tailored ORM framework for SMMEs to manage operational risk exposures from future disasters like COVID-19. A simplified approach was proposed, consisting of three stages: risk identification or disaster preparedness, risk analysis or disaster learning and risk treatment or building enterprise resilience. The framework was empirically tested on data from 208 tourism industry SMMEs using a quantitative research approach. Correlation analysis, structural equation modelling (SEM) and analysis of variance (ANOVA) tests were used to assess the framework’s applicability to businesses of different ages, sizes and subsectors. Results from correlations and SEM confirmed the proposed ORM framework’s effectiveness in explaining disaster preparedness, learning and resilience for SMMEs. Additionally, ANOVA results showed the framework was equally applicable across business subsectors, but across business age and size, it was not equally applicable. Larger and older businesses were able to implement disaster learning and resilience better than younger and less resourced businesses.

**Contribution:**

This research advances ORM within tourism SMMEs, proposing a simplified process validated by empirical findings demonstrating its effectiveness in proactive risk management and resilience during disaster incidents.

## Introduction

The COVID-19 pandemic severely impacted the global tourism sector, causing economic distress emanating from travel restrictions (Nhamo, Dube & Chikodzi 2020). At the business level, the effects were especially devastating for those reliant on public spaces such as restaurants (Toanoglou, Chemli & Marco 2022). Airlines, travel agencies and hospitality services experienced abrupt revenue losses that resulted in South African low cost carriers like Mango Airlines filing for bankruptcy and hotels seeing a downfall in occupancy (Maroof, Naz & Jawad 2021; Smith 2022). While the cascading financial losses from the pandemic were widespread (Nicola et al. 2020), post-lockdown recovery revealed the importance of solid resilience strategies that incorporate the potential effects of natural hazards (Androniceanu 2017; Bartik et al. 2020; Çelik & Ataç 2021).

As the occurrence of COVID-19 was novel, very few existing risk-management frameworks were prepared for such a disaster on a global scale (Harel 2021; Sharma, Thomas & Paul 2021). As a result, the pandemic’s effects were severe for all types of exposed businesses. To date, there are still limited empirical studies that have investigated the extent to which natural hazards have resulted in the revision of risk-management processes, especially within SMMEs. A question hence arises: what modifications can be made to the existing operational risk management (ORM) process for SMMEs to provide proactive action against potential risks of the magnitude of the COVID-19 pandemic? This paper seeks to develop and evaluate a tailored ORM framework for SMMEs to manage operational risk exposures caused by natural hazards of a magnitude similar to COVID-19. We draw from a current understanding of the ORM framework and an empirical study of SMMEs in the South African tourism sector to suggest and evaluate the applicability of a simplified framework for ORM for resource-limited small businesses.

## Literature review

Operational risk, as defined by the Basel Committee on Banking Supervision, refers to losses arising from internal processes, human error, system failures or external events (Strzelczak 2008). Notable examples of such events include the 2008/2009 Global Financial Crisis and the COVID-19 pandemic, which underscore the unpredictable nature and significant impact of operational risk events (Coleman 2011). This type of risk encompasses a wide range of potential issues such as fraud, legal risks, physical or environmental risks and technological failures (Torre-Enciso & Barros 2013). Aloqab, Alobaidi and Raweh (2018) note that unlike market or credit risks, operational risk is inherently tied to the operational activities of organisations, which makes it a critical area of focus for risk-management strategies. The evolution of risk management began in the banking sector where the Basel Accords provide guidelines for ORM in banking (Quillin 2008). Basel 1 initiated the focus on capital adequacy primarily addressing credit risk, while subsequent accords like Basel II and III expanded to incorporate operational risk and liquidity management (Slovik 2011). Despite their banking sector origins, the Basel Accords provide core principles adaptable to businesses of all sizes and across various industries, including tourism sector SMMEs (Basel Committee on Banking Supervision 2011). Integrating the Basel Committee’s principles for sound ORM with empirical insights on turnaround strategies post-COVID-19 forms our basis for the proposition of an ORM framework to enhance the resilience of tourism sector SMMEs.

A fundamental small business problem is resource limitation, which makes it challenging for SMMEs to implement legacy ORM frameworks. Scholars emphasise the benefits of robust ORM systems in reducing losses and enhancing shareholder value (Hanggraeni et al. 2019). Small businesses typically face risks categorised into opportunity-based, uncertainty-based and hazard-based risks, each requiring specific mitigation approaches such as upside-downside analysis, disaster planning and workplace safety improvements. The typical ORM process for businesses derived from the International Standardization Organization (ISO) 31000 framework involves five steps: establishing context, identifying risks, analysing risks, evaluating risk levels and treating risks through avoidance, mitigation or transfer strategies (Ekwere 2016; Verbano & Venturini 2013). While some risk-management measures may not directly apply to certain disaster situations like pandemics, the comprehensive ORM approach encompasses various sources of risk beyond natural hazards, including financial, marketing and legal risks. This highlights the importance of proactive risk management for small businesses (Walker et al. 2020).

While exploring literature on ORM within SMMEs, it becomes evident that these businesses face significant challenges in implementing robust risk-management practices. Often, risk management in these businesses is informal, leaving them vulnerable to severe consequences like revenue loss and closure (Qubtan et al. 2021). Falkner and Hiebl (2015) highlight disparities in the implementation of risk-management steps, with project management often receiving more emphasis than day-to-day activities because of limited knowledge among employees and management. Sarmiento et al. (2016) note that, despite significant investments in their businesses, small business owners often neglect disaster planning. Sukumar, Edgar and Grant (2011) argue that resource constraints hinder the adoption of comprehensive ORM practices. However, simplified frameworks, such as those suggested by Marcelino-Sádabaa et al. (2014), offer practical solutions for SMMEs with limited technical expertise. Risk-treatment options, including insurance and supplier agreements, also play a key role in mitigating operational risks within SMMEs.

The influence of business factors on risk-management practices is another important area of research. Kruger (2021) demonstrates how factors such as organisational size, owner age and experience shape risk-management attitudes. These factors highlight the need for tailored approaches to risk management by responding to individual business factors. However, knowledge gaps and difficulties in risk analysis persist especially for highly resource-deficient SMMEs (Falkner & Hiebl 2015; Zoghi 2017). Despite these challenges, there is growing recognition of the need for structured risk management to improve risk awareness and preparedness in SMMEs, aligning organisational goals with employee understanding (Henschel 2008; Kokot-Stępień 2023; Prioteasa et al. 2020) inspired by global risk events such as COVID-19.

Recent studies on the impact of COVID-19 on risk management and SMME turnaround strategies reveal varying levels of resilience among small businesses (Chang, McAleer & Wong 2020; Ekechi et al. 2024; Grondys et al. 2021; Mthiyane, Van-der-Poll & Tshehla 2022). While financial and market risks dominate concerns, operational risks remain critical mostly in terms of resource utilisation and innovation. However, the varying levels of effectiveness of these risk management approaches show the need for strategies tailored to organisational characteristics and sector-specific challenges. The literature on developing tailored ORM frameworks for SMMEs is limited, especially regarding disaster risk management, highlighting the necessity of our proposed framework.

This research’s theoretical framework is underpinned by two major conceptualisations of ORM. Firstly, we draw lessons from the Basel Committee’s principles for sound risk management to conceptualise and evaluate our proposed framework. In particular, principle 1 (risk management culture), principle 2 (integrating risk management with overall business processes), principle 6 (risk identification and assessment), principle 8 (monitoring and reporting) and principle 11 (business continuity) were highly instrumental in shaping our conceptualisation of ORM at the SMME level. Secondly, the research is modelled after the ISO 31000 process for risk management (Ekwere 2016) which entails establishing the context, identifying risks, analysing risks, evaluating and treating risks. We argue that while this process is well-established and provides a comprehensive framework for business’ risk management, its implementation can be complex and unfeasible for resource-starved small businesses. Unlike larger organisations, SMMEs lack the resources and expertise to implement established risk-management frameworks effectively. As a result, we propose a tailored ORM framework which aims to address gaps in risk-management practices and support SMME resilience during crises like COVID-19. The central premise behind our proposed framework is the simplification of ORM processes to ensure SMME’s comprehensive management of risk without the use of complex systems, in line with Falkner and Hiebl (2015), Naude and Chiweshe (2017) and Mthiyane et al. (2022).

## Proposed operational risk management framework

**FIGURE 1:**
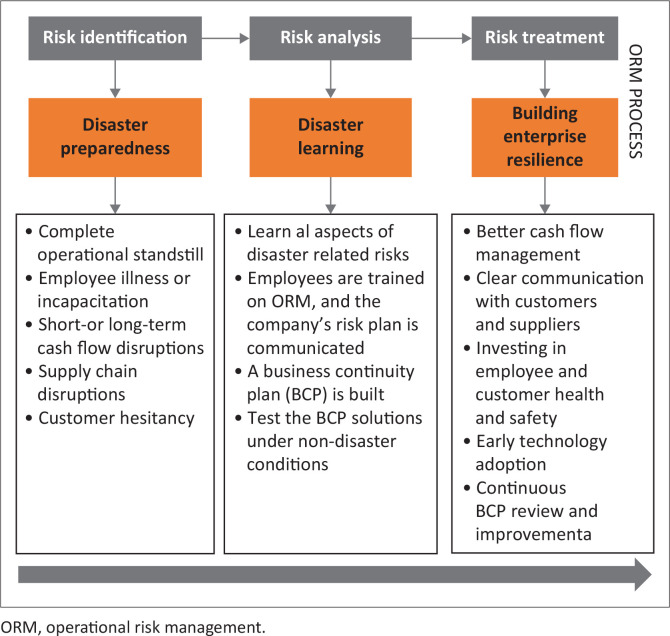
Proposed disaster operational risk management framework for SMMEs in the tourism sector.

Figure 1 shows the proposed framework for ORM within the tourism sector SMMEs during disaster incidents, separated into three stages. The three stages include risk identification, where pandemic-type risks like employee incapacitation and cash flow disruptions are highlighted, informed by disaster learning and business continuity planning. Risk analysis entails learning from past disasters, with an emphasis on business continuity planning and clear communication of risk stances to employees. Risk-treatment strategies, such as improved cash flow management and adoption of new technologies, aim to build enterprise resilience. While not a one-size-fits-all solution, these strategies provide a foundation for creating resilient SMMEs post-disaster (Abdin 2020; Jedynak & Bąk 2021; Prasad et al. 2015).

In addition to proposing the framework, our paper also sought to evaluate the proposed framework using data collected from a sample of tourism sector SMMEs in South Africa. Specifically, we sought to (1) assess the extent to which the developed ORM framework matches existing ORM practices within SMMEs in South Africa and (2) determine the influence of the business subsector, years of operation and estimated annual revenue on ORM practices within tourism sector SMMEs in South Africa.

## Research methods and design

### Study design

Our research, which is grounded in the positivist philosophy, employed a survey strategy for the collection of data from multiple sources, as well as the use of statistical inferences to obtain representative results. We employed a quantitative and deductive research approach, allowing us to benefit from existing knowledge before formulating the study framework, then evaluate the proposed framework by explaining the causal relationships between variables and concepts. The survey comprised 208 tourism sector SMMEs across South Africa, and the study questionnaire was deployed using Google Forms, while the link for completion was distributed using emails.

### Study population and sampling strategy

The study population consisted of 500 tourism sector small businesses listed in a database acquired from a research company based in Pretoria, South Africa. The population of small businesses in tourism was selected because of the common lack of proper risk-management processes within SMMEs, which was exacerbated by the pandemic. Employing a stratified random sampling method, the population was divided into subsectors like aviation, travel and tours and accommodation to ensure diversity in the distribution of participants. From the population, a total of 208 responses were completed in good order for analysis and were used in the research.

### Data collection

The research procedure encompassed the following stages: an extensive literature review to establish constructs and hypotheses, the design of a questionnaire aligned with the proposed ORM framework, obtaining ethical clearance from the University of South Africa and the collection of data using a structured questionnaire. The questionnaire employed contained four main sections: Section A measured the disaster preparedness variable, Section B measured the disaster learning variable, Section C assessed the enterprise resilience variable and Section D collected business demographic factors (age, subsector and annual revenue). The survey items for the scale were scored on a five-point Likert-type response format ranging from 1 = ‘strongly disagree’ to 5 = ‘strongly agree’. To enhance reliability and validity, the questionnaire was pilot-tested with 10 participants, and pilot results were used to revise questions for ease of response.

### Data analysis

We employed the Statistical Package for Social Sciences (SPSS) version 25 and R programming language for the quantitative analysis to assess ORM practices within the tourism sector SMMEs post-COVID-19. Descriptive statistics were used to present frequencies and central tendency measures, while inferential statistics included correlation analysis and structural equation modelling (SEM) to validate the proposed ORM framework. We sought to examine the linear associations between disaster preparedness, disaster learning, and enterprise resilience with correlation analysis and employ SEM to explore the sequence of relationships among these variables. Additionally, we utilised analysis of variance (ANOVA) tests and a non-parametric test (Kruskal–Wallis H) to evaluate differences in ORM practices based on the factors of SMME subsector, years of operation and estimated annual revenue.

### Ethical considerations

Ethical clearance to conduct this study was obtained from the University of South Africa College of Economic and Management Sciences_ERC Finance, Risk Management & Banking (No. 1462). The study adhered to ethical guidelines and protocols to ensure the rights and integrity of participants’ data and adhered to established protocols such as the *Protection of Personal Information Act* (POPIA). A tick box to affirm participation after reading all information about the study and participants’ rights was included on the first page of the questionnaire online, and participants could not sign it directly. Moreover, the information gathered was kept private and utilised exclusively for the research project. No personal data about the participants, including names or ages, were gathered, and the study’s conclusions were derived from a collation of data instead of individual responses.

## Results

### Extent to which the proposed framework matches existing operational risk management practices

The first objective of this paper was to evaluate the extent to which the developed ORM framework matches existing ORM practices within SMMEs in South Africa. In this section, we present and discuss the results of the evaluation, which was conducted using correlation analysis and SEM to assess the relationships between the three elements of the proposed ORM framework.

#### Descriptive statistics

Table 1 shows the distribution of participants by various demographic factors. In terms of subsector, most of the participants (35.6%) were from the travel and tours subsector, followed by accommodation providers (26.8%), restaurant and catering (22.4%) and aviation businesses (15.2%). Regarding the title/designation of participants, almost half of the participants (47.6%) were owners or directors of their businesses, followed by managers (28.8%), risk officers (17.8%) and staff (5.8%). For the distribution by business age, the majority of participants (40.4%) had businesses that were 5–10 years old, while (30.3%) had businesses that were more than 10 years old, 14.4% were less than 3 years old and 14.9% were 3–5 years old (14.9%). Lastly, by annual revenue, most participants’ businesses (41.8%) had an annual revenue of greater than R5 million but less than or equal to R15 million, while others (32.2%) had an annual revenue of more than R15 million but less than or equal to R40 million, and the least participants (26.0%) had an annual revenue of less than or equal to R5 million.

**TABLE 1a:** Descriptive statistics.

Distribution by subsector	Percentage of participants	Designation of respondents in the organisation	Percentage of participants
Aviation business	15.2	Owner or director	14.4
Travel and tours	35.6	Manager	14.9
Accommodation	26.8	Risk officer	40.4
Restaurant and catering	22.4	Staff	30.3

**TABLE 1b:** Descriptive statistics.

Years of operation	Percentage of participants	Estimated annual revenue	
		Revenue ranges	Percentage of participants
< 3	47.6	≤ R5 million	26
3–5	28.8	≤ R15 million	42
5–10	17.8	≤ R40 million	32
> 10	5.8	-	-

#### Correlation analysis

With correlation analysis, we assess the linear associations between components of the proposed ORM process to determine whether disaster preparedness activities indeed relate to disaster learning and enterprise resilience activities, as hypothesised. Our analysis sought to test the following hypothesis:

**Hypothesis 1:** There are no significant associations between disaster preparedness, disaster learning and enterprise resilience.

The analysis revealed significant positive correlations between disaster preparedness and learning (Pearson’s *r* = 0.600, *p* = 0.001), disaster learning and enterprise resilience (Pearson’s *r* = 0.647, *p* = 0.001) and disaster preparedness and business resilience (Pearson’s *r* = 0.474, *p* = 0.001). This implies that businesses with higher preparedness levels are more inclined to engage in learning and resilience-building practices.

The analysis hence supports the proposed framework, showing the importance of preparedness, learning and resilience as part of the ORM process for small businesses. Our findings also support the hypotheses advanced by Abdin (2020) and Prasad et al. (2015) and highlight the importance of comprehensive risk identification before risk analysis, as suggested by Ekwere (2016) and Verbano and Venturini (2013). Moreover, the results align with ISO 31000 standards, which prioritise risk identification, followed by risk assessment in the ORM process, as noted by Ekwere (2016) and Chang et al. (2020). We also note SMMEs’ inclination to invest in resilience measures tailored to their specific risk concerns (arising from disaster learning), an approach which corroborates the views of Grondys et al. (2021) and Marcelino-Sádabaa et al. (2014). This approach, consistent with our proposed framework, allows businesses to allocate resources effectively to mitigate identified risks (Guo 2015; Marcelino-Sádabaa et al. 2014).

#### Structural equation modelling

We employed SEM to examine the relationships between the three key components of the proposed ORM framework: preparedness, learning and resilience. Specifically, we sought to test the following hypotheses:

**Hypothesis 2:** Disaster preparedness does not lead to disaster learning.

**Hypothesis 3:** Disaster learning does not lead to enterprise resilience.

**Hypothesis 4:** Enterprise resilience does not lead to disaster preparedness.

In the SEM framework, the latent variables of preparedness, learning and resilience were constructed using their five respective measured components. The estimates for these components indicate the factor loadings, showing how each measured component contributed to the latent variable.

**FIGURE 2:**
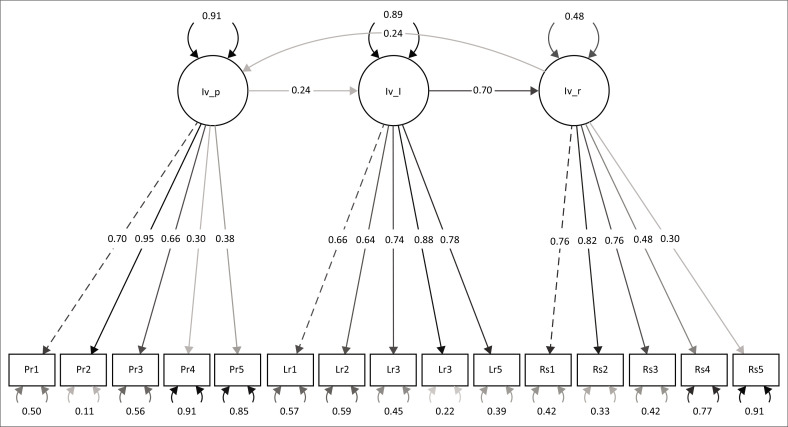
Structural equation modelling of the relationships between preparedness, learning and resilience.

For example, in Figure 2, in the case of preparedness, ‘Preparedness2’ contributes the most (with a factor loading of 0.95), followed by ‘Preparedness1’ (with a factor loading of 0.70). Moreover, a significant positive relationship was found between learning and preparedness (0.225), indicating that disaster learning had a positive influence on preparedness. Similarly, the relationship between learning and resilience was found to be strong (0.796), supporting the hypothesis that disaster learning positively affects enterprise resilience. However, the relationship between preparedness and resilience (0.173) was weaker and not statistically significant (*p* = 0.138).

The SEM results partially support our proposed ORM framework, confirming the sequential relationship between disaster preparedness and learning and learning and enterprise resilience, but not between enterprise resilience and disaster preparedness. The weak positive relationship between enterprise resilience may suggest that the proposed framework works more as a linear process rather than a full circle. These findings are consistent with the positive correlations observed in the correlation analysis. However, while the correlation between preparedness and resilience was positive, it was weaker and statistically insignificant in the SEM framework. The SEM results also support existing knowledge of the relationships between risk identification, analysis and treatment (Chang et al. 2020). However, the weak influence of enterprise resilience appears to contradict the existing ISO 31000 framework, which recognises that ORM is a circular process (Ekwere 2016; Verbano & Venturini 2013). This mismatch may, however, be understood to be minimal, given how both correlation analysis and SEM still confirmed the existence of the relationship, although it is weaker in the SEM results.

### Influence of SMME business factors on operational risk management practices

The second objective was to determine the influence of the business factors on ORM practices of the tourism sector SMMEs in South Africa. We employed ANOVA and Kruskal–Wallis H tests to evaluate the extent to which our framework applies to different types of SMMEs and to identify to which business types our framework applies strongest. Table 1 summarises the relationships between the SMMEs’ subsectors, ages and annual revenues as revealed by statistical tests, as well as their implementation of ORM practices. The following hypotheses are tested:

**Hypothesis 5:** There is no relationship between SMMEs’ subsector and their ORM practices.

**Hypothesis 6:** There is no relationship between SMMEs’ age and their ORM practices.

**Hypothesis 7:** There is no relationship between SMMEs’ size and their ORM practices.

Regarding the SMME subsector, the ANOVA and Kruskal–Wallis tests indicate that the business operation subsector did not significantly influence disaster preparedness and learning practices, but it substantially impacts enterprise resilience activities. While preparedness and learning practices were consistent across subsectors, we found notable differences in resilience practices, with the aviation subsector showing the highest mean rank, followed by accommodation providers, restaurant and catering and travel and tours. This suggests that our ORM framework’s resilience practices were more applicable to aviation SMMEs and less so to others, a reflection of the aviation industry’s swift disaster recovery and the varying levels of preparedness among subsectors (Maroof et al. 2021).

As in Table 2, for business age, the ANOVA and Kruskal–Wallis tests indicate that business age significantly influences disaster preparedness, learning about disaster-related risks and enterprise resilience, with older businesses demonstrating higher levels of these ORM practices. Specifically, businesses with more experience, ranging from 5 to over 10 years, exhibited greater preparedness, learning and resilience compared to newer businesses. This underscores the importance of experience in effectively implementing ORM strategies. Our results also corroborate existing literature emphasising the link between risk management capability and business experience (Abdin 2020; Ayandibu & Houghton 2017). Additionally, Kruger (2021) highlights the significance of factors such as owner age, education level and years of experience in shaping ORM practices, which are in alignment with the results of this paper.

**TABLE 2:** Application of proposed operational risk management practices by different business factors.

Components	ANOVA test result		Kruskal–Wallis test result		Decision
	*F*	*p*	*H*(*3*)	*p*	
Subsector					
Preparedness	2.734†	0.055	7.054	0.070	Do not reject
Learning	1.911†	0.129	7.464	0.058	Do not reject
Resilience	5.496†	0.001	15.596	0.001	Reject
Age of business					
Preparedness	7.640‡	0.000	14.563	0.002	Reject
Learning	35.059‡	0.000	60.280	0.000	Reject
Resilience	39.680‡	0.000	72.078	0.000	Reject
Annual revenue					
Preparedness	5.342‡	0.059	6.296	0.601	Do not reject
Learning	15.988‡	0.000	27.330	0.000	Reject
Resilience	19.945‡	0.000	32.150	0.000	Reject

ANOVA, analysis of variance.

† , F(3, 204);

‡ , F(3, 207).

Concerning the annual revenue, results reveal no significant difference in disaster preparedness practices across SMME size groups (The stated value (*p* > 0.05), suggesting our framework’s applicability regardless of business size. However, substantial differences were observed in disaster learning and resilience practices (*p* < 0.05), indicating varied applicability based on SMME size. This highlights the need to consider financial capacity when implementing ORM practices, in alignment with the literature on the influence of resources on risk management (Abdin 2020; Falkner & Hiebl 2015; Kokot-Stępień 2023). While larger SMMEs are more likely to invest in resilience (Naude & Chiweshe 2017), microenterprises face disproportionate risks because of resource limitations, which ironically necessitates their need to enhance disaster learning and resilience (Kruger 2021). While Androniceanu (2017) suggests that entrepreneurs can effectively analyse risks irrespective of business size, our findings indicate otherwise, supporting the need for ORM frameworks tailored to SMMEs (Henschel 2008; Marcelino-Sádabaa et al. 2014).

### Limitations

Primarily focusing on the tourism sector, the findings of this paper might not apply to SMMEs in other industries. Hence, there is a need for future research targeting different sectors. Additionally, our proposed ORM framework lacks universality because of variations among businesses, especially in extremely resource-constrained businesses like sole traders, which we found to be hardly implementing the equivalent to our simplified ORM process. Furthermore, our research overlooks individual-level factors within SMMEs, such as leadership styles and organisational culture, which potentially influence ORM practices. Future studies exploring these internal business dynamics could enrich our understanding and guide interventions for the most tailored ORM frameworks for SMMEs.

## Discussion

This study contributes to the understanding of ORM practices in South African tourism SMMEs, addressing challenges like limited resources and employee know-how through a simplified ORM process. The proposed framework, developed from literature and post-COVID-19 disaster resilience strategies, has implications for practice as it offers a structured approach for risk identification, analysis and treatment, specifically targeting disaster-type risks. Moreover, we highlight the influence of business factors like subsector, age and revenue on ORM practices, suggesting a need for tailored frameworks applicable to businesses of multiple characteristics. The findings offer valuable insights for policymakers, such as the Department of Small Business Development in South Africa, aiming to support SMMEs. By understanding the specific ORM challenges faced by small businesses, policies can be designed to provide targeted support, such as financial aid, training programmes and resources to enhance disaster preparedness and resilience.

There are, however, still emergent avenues for future research into enhancing SMME disaster resilience. Firstly, there is a need to refine our ORM framework to better suit diverse SMME characteristics, especially those with extreme resource scarcity. Secondly, future research could explore integrating emerging technologies like AI and data analytics into ORM practices to improve disaster preparedness. Additionally, micro-level empirical research within SMMEs could discover the role of individual-level factors in ORM implementation, informing modified frameworks for businesses of diverse characteristics.

## Conclusion

This paper aimed to develop and evaluate a tailored ORM framework for SMMEs in the South African tourism sector in response to disasters similar to the COVID-19 pandemic. From a review of the literature, we established the challenges faced by SMMEs which inhibit their application of established legacy ORM frameworks, primarily because of their limited expertise and resources. Subsequently, we propose an ORM framework anchored on simplicity by focusing on disaster preparedness, learning and resilience. The proposed ORM process was empirically tested on data collected from 208 small businesses in the tourism industry using quantitative research. Our main findings underscore the framework’s effectiveness in explaining disaster preparedness, learning and resilience for SMMEs, with some variations across business subsectors, ages and annual revenues. Based on the findings, we highlight the importance of proactive risk identification, continuous learning and adaptive resilience strategies for SMMEs. The adoption of simplified risk-management terminology and networking is also crucial for under-resourced SMMEs unable to formalise risk management. Future research can focus on refining the framework, exploring its application across sectors and considering technological impacts on ORM practices.
